# Supplementary material for: SPX-related genes regulate phosphorus homeostasis in the marine phytoplankton, Phaeodactylum tricornutum
Source: Commun Biol. 2021 Jun 25;4:797. doi: 10.1038/s42003-021-02284-x (PMC8233357; doi:10.1038/s42003-021-02284-x)
Supplement: Supplementary file 2 — Supplementary Information [file 42003_2021_2284_MOESM2_ESM.pdf]

Supplementary Information

SPX-related genes regulate phosphorus homeostasis in the marine phytoplankton, *Phaeodactylum tricornutum*

<u>Content:</u> Supplementary figure (1 : 3) and Supplementary table (1 : 10)

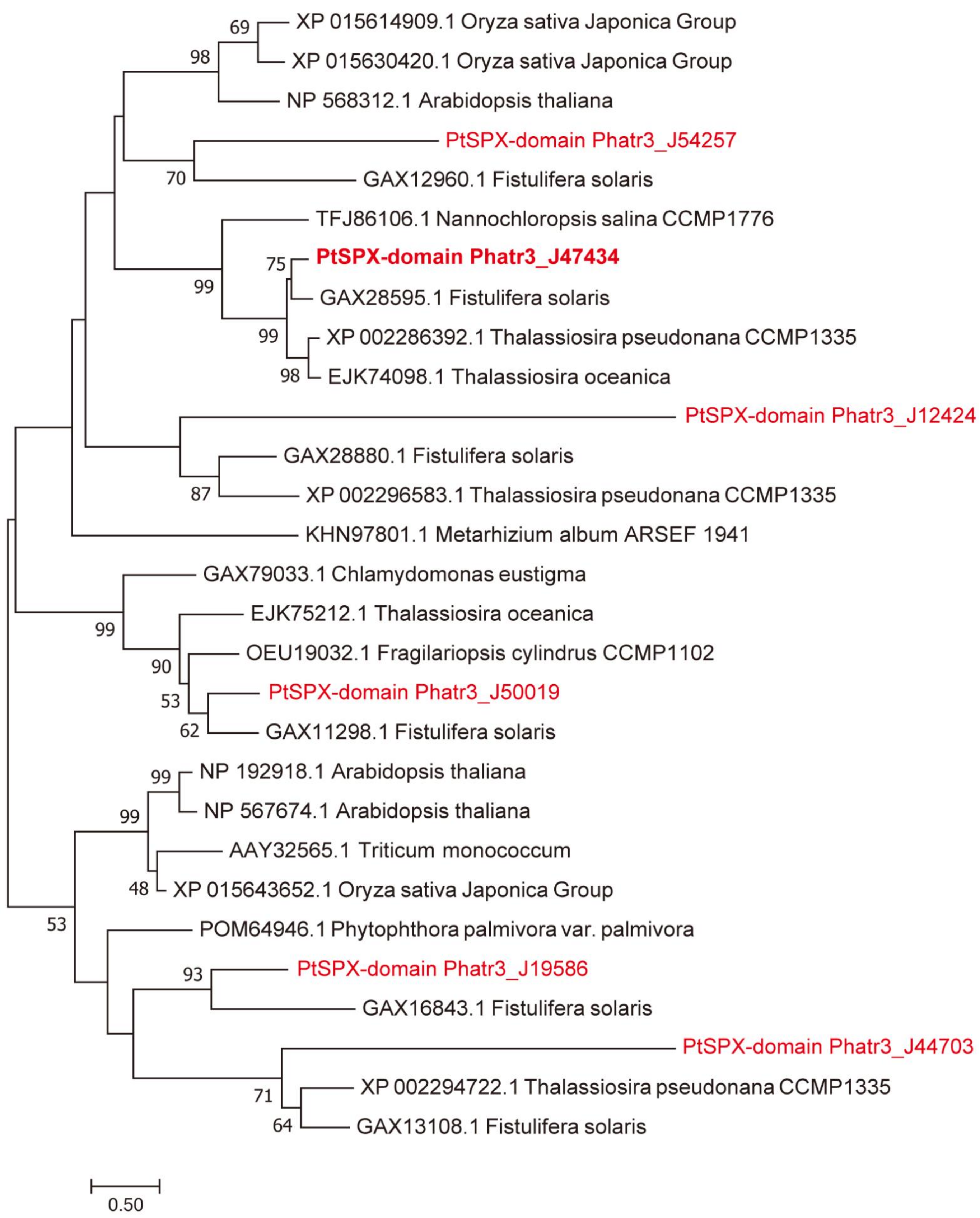

Supplementary Figure 1. Maximum likelihood tree based on amino acid sequences of SPX domains previously reported in plants (*Arabidopsis*, Oryza, *Triticum*) and those identified in this study from algae. The SPX domain-containing genes in *P. tricornutum a*re marked in red and the bold-typed one (Phatr3_J47434) was mutated in our study. Values at nodes are bootstrap support values (only those > 50% are shown). Scale bar depicts substitution rate per amino acid residue.

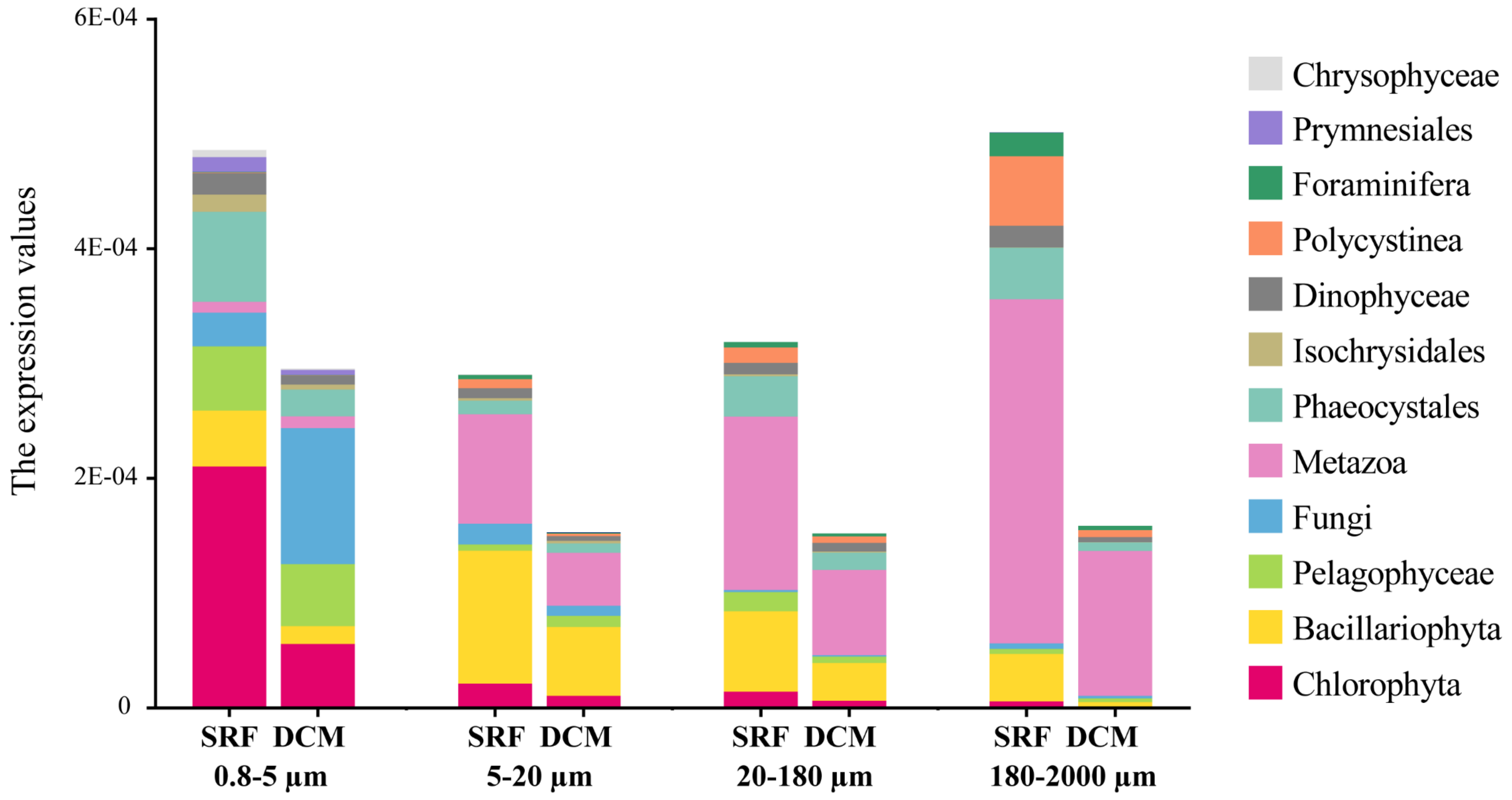

Supplementary Figure 2. Expression of the SPX domain-containing genes in different lineages and different size fractions. The expression values were computed as RPKM. SRF, subsurface; DCM, deep chlorophyll maximum layer. The SRF layer and DCM layer are displayed on the left and right, respectively. The color block depicts the expression of certain lineage in different size fractions (0.8-5 µm, 5-20 µm, 20-180 µm, and 180-2000 µm).

Supplementary Figure 3. Uncropped gel image

Fig. 1d

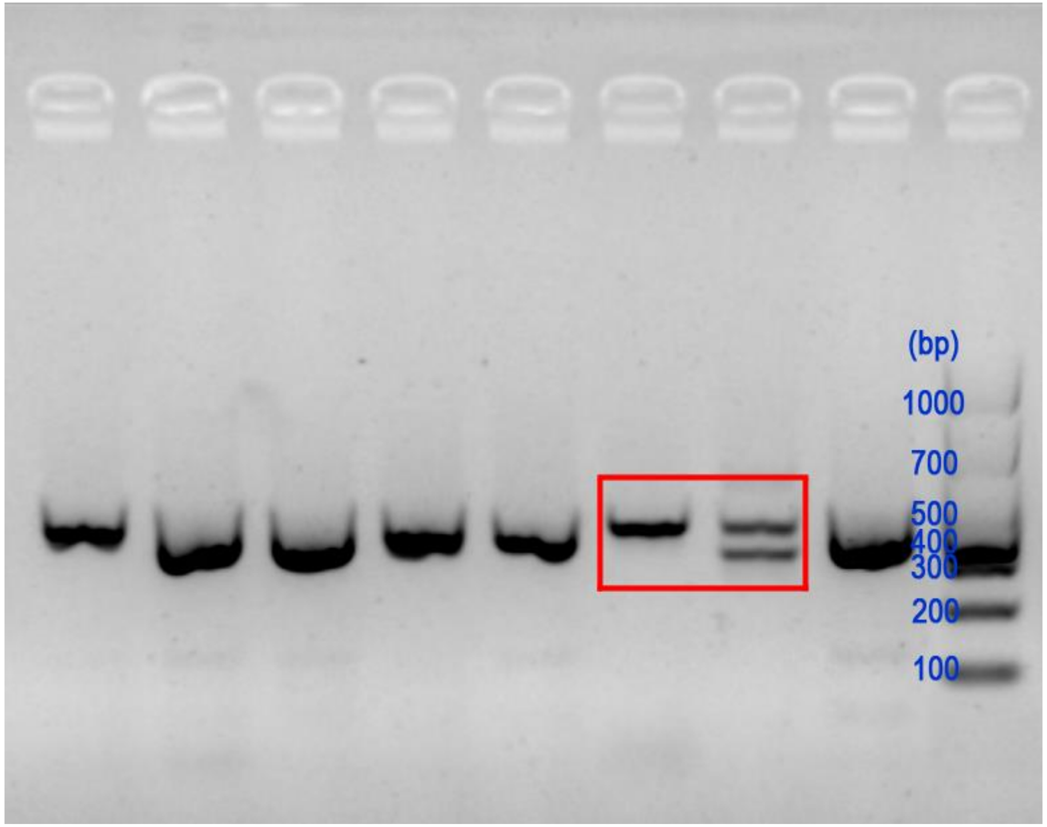

Supplementary Figure 3. Uncropped gel image corresponding to Fig. 1d

Supplementary Table 1. SPX domain-containing genes detected from the transcriptomes of wild type *P. tricornutum* and their expression changes between P-stress (P-) and P-replete (P+) growth conditions.

GeneID	Gene type	log_2 FC (P-/P+)[b]	adjusted p-value	Up/Down	Domains
Phatr3_J50019	PtVtc4	4.24	0	Up*	SPX-VTC
Phatr3_J19586	PtVpt1	4.09	0	Up*	SPX-MFS
Phatr3_J47434[a]	PtSPX	3.06	0	Up*	SPX
Phatr3_J44703	PtSPX1	-0.51	3.49E-15	Down	SPX-MFS
Phatr3_J54257	PtSPX2	-0.56	0.055	-	SPX-Cytidylyltransferase like
Phatr3_J12424	PtSPX3	-0.36	1.93E-11	Down	SPX-EXS

[a] Genes chosen for CRISPR/Cas9 knockout in this study.

[b] Log_2 fold change between P- and P+ conditions based on RNA-seq data. Each value is the mean from three biological replicates.

* Significance of change is set at the absolute value of log_2 FC > 1 and adjusted p-value < 0.05 in this study.

[-] No change in transcriptional level.

Supplementary Table 2. Transcriptome mapping statistics in *P. tricornutum*.

Library	Total raw reads[a] (M)	Total clean reads[b] (M)	Clean reads ratio (%)	Mapping ratio (%)	Uniquely mapping ratio[c] (%)
*m*SPX15-3_P+_1	24.14	24.1	99.85	93.24	83.27
*m*SPX15-3_P+_2	20.63	20.59	99.82	93.42	83.29
*m*SPX15-3_P+_3	24.14	24.1	99.86	93.29	83.12
*m*SPX15-3_P-_1	24.14	24.1	99.87	94.08	85.35
*m*SPX15-3_P-_2	24.14	24.1	99.87	93.93	85.14
*m*SPX15-3_P-_3	24.14	24.1	99.87	93.96	85.25
WT_P+_1	24.14	24.1	99.87	94.18	84.55
WT_P+_2	24.14	24.1	99.86	94.1	84.65
WT_P+_3	24.14	24.1	99.85	94.17	84.80
WT_P-_1	24.14	24.1	99.85	94.44	85.78
WT_P-_2	24.14	24.09	99.85	94.27	85.47
WT_P-_3	24.14	24.1	99.87	94.17	85.26

[a] The number of reads before filtering, Unit: Mb.

[b] The number of reads after filtering, Unit: Mb.

[c] The percentage of reads that map to only one location of reference.

Supplementary Table 3. Relative expression of AP genes in *m*SPX determined by RNA-seq under P-replete (P+) and P-stress (P-) conditions.

Gene ID	Gene type	*m*SPX P+/WT P+			*m*SPX P-/WT P-		
		log_2 FC[a]	adjusted p-value	Up/Down	log_2 FC[a]	adjusted p-value	Up/Down
Phatr3_J49678	PhoA	3.18	5.82E-292	Up*	0.91	3.87E-21	Up
Phatr3_J45959	PhoD	-0.66	3.50E-06	Down	0.79	6.61E-09	Up
Phatr3_J45757	PhoD	2.56	2.63E-103	Up*	0.89	1.60E-58	Up
Phatr3_J39432	PhoD	4.92	1.51E-287	Up*	1.41	5.24E-144	Up*
Phatr3_J47612	AP[b]	1.47	4.74E-50	Up*	0.75	4.46E-41	Up
Phatr3_J47869	PhoA[aty]	1.93	7.36E-148	Up*	1.20	2.28E-67	Up*
Phatr3_J48970	PhoD	-0.13	0.580	-	-0.06	0.775	-
Phatr3_J45174	PhoD	-0.91	1.61E-07	Down	-0.47	0.001	Down

[a] Log_2 fold change between P- and P+ conditions based on RNA-seq data. Each value is the mean from three biological replicates.

[b] Undetermined type of AP

[aty] depicts an atypical subtype of PhoA.

* Significance of change is set at the absolute value of log_2 FC > 1 and adjusted p-value < 0.05 in this study.

[-] No change in transcriptional level.

Supplementary Table 4. Relative expression of phosphate transporter genes in *m*SPX determined using RNA-seq under P-replete (P+) and P-stress (P-) conditions.

Gene ID	Gene type	*m*SPX P+/WT P+			*m*SPX P-/WT P-		
		log_2 FC[a]	adjusted p-value	Up/Down	log_2 FC[a]	adjusted p-value	Up/Down
Phatr3_J33266	PtNap$_i$1	0.07	0.825	-	0.29	0.007	Up
Phatr3_J40433	PtNap$_i$2	0.42	1.48E-08	Up	-0.21	2.09E-04	Down
Phatr3_J47239	PtNap$_i$3	6.35	0	Up*	1.60	1.55E-69	Up*
Phatr3_J47667	PtNap$_i$4	0.77	2.05E-15	Up	0.21	1.40E-04	Up
Phatr3_J49842	PtNap$_i$5	-0.25	0.074	-	-0.06	0.717	-
Phatr3_J47666	PtNap$_i$6	0.89	2.39E-18	Up	0.34	3.80E-11	Up
Phatr3_J23830	PtPho4	1.31	3.33E-58	Up*	0.81	3.69E-12	Up
Phatr3_J39515	PtIPT	1.20	6.19E-29	Up*	0.97	4.05E-30	Up
Phatr3_J22315	PtMPT	2.05	1.61E-12	Up*	1.87	1.76E-64	Up*
Phatr3_J19586	PtVpt1	1.02	7.22E-12	Up*	0.54	1.42E-13	Up
Phatr3_J17265	PtHp$_i$1	0.15	0.519	-	-0.19	0.068	-
Phatr3_Jdraft462	PtHp$_i$2	-0.29	0.169	-	-0.28	0.083	-

[a] Log_2 fold change between P- and P+ conditions based on RNA-seq data. Each value is the mean from three biological replicates.

* Significance of change is set at the absolute value of log_2 FC > 1 and adjusted p-value < 0.05 in this study.

[-] No change in transcriptional level.

Supplementary Table 5. The expression of phospholipid degradation related genes in *m*SPX determined by RNA-seq under P-replete (P+) and P-stress (P-) conditions.

GeneID	*m*SPX P+/WT P+			*m*SPX P-/WT P-			Description
	log_2 FC[a]	adjusted p-value	Up/Down	log_2 FC[a]	adjusted p-value	Up/Down	
Phatr3_J52110	1.40	3.85E-18	Up*	1.09	4.02E-27	Up*	ethanolamine-phosphate phospholyase
Phatr3_J49693	2.45	2.61E-81	Up*	-0.23	0.011	Down	glycerophosphoryl diester phosphodiesterase
Phatr3_J44900	1.66	4.20E-23	Up*	1.81	1.12E-95	Up*	glycerophosphoryl diester phosphodiesterase
Phatr3_J32057	2.29	3.14E-185	Up*	1.21	7.33E-88	Up*	glycerophosphoryl diester phosphodiesterase
Phatr3_J14785	0.72	0.529	-	1.36	6.24E-07	Up*	peroxiredoxin 6, 1-Cys peroxiredoxin
Phatr3_J43665	1.60	2.23E-36	Up*	0.83	1.10E-18	Up	PLC-like phosphodiesterase
Phatr3_EG00718	2.15	2.33E-108	Up*	1.18	1.18E-76	Up*	acyl transferase/acyl hydrolase/lysophospholipase
Phatr3_J49702	0.88	7.95E-16	Up	1.18	1.49E-40	Up*	phospholipid--sterol O-acyltransferase isoform X4
Phatr3_J42871	1.44	6.18E-27	Up*	1.46	7.35E-119	Up*	phospholipid methyltransferase
Phatr3_J40163	1.88	3.24E-48	Up*	1.76	2.22E-59	Up*	ethanolamine-phosphate cytidylyltransferase
Phatr3_J55111	1.25	1.49E-36	Up*	0.51	2.90E-10	Up	phospholipid scramblase

[a] Log_2 fold change between P- and P+ conditions based on RNA-seq data. Each value is the mean from three biological replicates.

* Significance of change is set at the absolute value of log_2 FC > 1 and adjusted p-value < 0.05 in this study.

[-] No change in transcriptional level.

Supplementary Table 6. Myb transcription factors (Myb TFs) identified and their expression changes in different comparisons.

Gene ID	TF name	WT P+/WT P-			*m*SPX P+/WT P+			*m*SPX P-/WT P-		
		log_2 FC[a]	adjusted p-value	Up/Down	log_2 FC[a]	adjusted p-value	Up/Down	log_2 FC[a]	adjusted p-value	Up/Down
Phatr3_EG02570	Pt_Myb1R_1a	0.21	0.003	Up	-0.23	0.094	-	0.04	0.731	-
Phatr3_EG01922	Pt_Myb1R_1b	0.86	1.99E-31	Up	-0.17	0.269	-	0.06	0.552	-
Phatr3_J45431	Pt_Myb1R_2	-0.57	1.65E-23	Down	0.03	0.873	-	0.39	8.98E-07	Up
Phatr3_J44256	Pt_Myb1R_3	-0.75	1.52E-21	Down	-0.09	0.722	-	0.85	1.67E-22	Up
Phatr3_J44331	Pt_Myb1R_4	1.09	9.45E-19	Up*	0.56	0.303	-	0.26	0.372	-
Phatr3_J47256	Pt_Myb1R_5	4.30	0	Up*	0.91	3.3E-10	Up	1.84	1.87E-131	Up*
Phatr3_J46535	Pt_Myb1R_6	0.07	0.313	-	-0.03	0.887	-	-0.14	0.056	-
Phatr3_J49838	Pt_Myb1R_7	-0.48	8.32E-10	Down	-0.25	0.109	-	-0.57	2.69E-06	Down
Phatr3_J50328	Pt_Myb1R_8	1.80	2.15E-66	Up*	-0.11	0.615	-	0.68	1.06E-08	Up

[a] Log_2 fold change between P- and P+ conditions based on RNA-seq data. Each value is the mean from three biological replicates.

* Significance of change is set at the absolute value of log_2 FC > 1 and adjusted p-value < 0.05 in this study.

[-] No change in transcriptional level.

Supplementary Table 7. The information of fatty acid biosynthesis genes in *m*SPX/WT comparisons under P+ and P- conditions of *P. tricornutum*.

Gene ID	*m*SPX P+/WT P+			*m*SPX P-/WT P-			Description	Symbol
	log_2 FC[a]	adjusted p-value	Up/Down	log_2 FC[a]	adjusted p-value	Up/Down		
Phatr3_EG01955	-1.71	1.26E-23	Down*	-3.36	8.07E-40	Down*	acetyl-coa carboxylase	ACC
Phatr3_J37652	-3.71	4.50E-26	Down*	-2.14	2.88E-02	Down*	malonyl-CoA:ACP transacylase	MAT
Phatr3_J37367	-1.53	5.38E-13	Down*	-1.50	6.29E-05	Down*	3-ketoacyl-ACP synthase II	KASII
Phatr3_J52648	-2.75	9.12E-27	Down*	-0.41	0.139	-	3-oxoacyl-[acyl-carrier-protein] synthase	KAR
Phatr3_J9709	-1.36	4.72E-15	Down*	-1.68	2.09E-11	Down*	acyl carrier protein	ACP
Phatr3_J10068	-1.87	7.17E-16	Down*	-1.42	0.072	-	enoyl-acp reductase	EAR
Phatr3_J55209	-2.80	3.61E-61	Down*	-0.37	8.12E-03	Down	biotin carboxylase	BC
Phatr3_J25769	-2.78	9.00E-20	Down*	-0.84	1.13E-05	Down	delta 12 fatty acid desaturase	FADS12
Phatr3_EG02619	-1.05	1.04E-07	Down*	-0.83	4.60E-06	Down	fatty acid desaturase	FADS
Phatr3_J55137	-1.34	1.11E-14	Down*	-0.47	1.12E-04	Down	acyl desaturase	FADS
Phatr3_J48423	-1.38	2.88E-16	Down*	-3.92	9.79E-27	Down*	precursor of desaturase omega-6 desaturase	FADS
Phatr3_J28797	-1.17	1.93E-06	Down*	-0.66	4.89E-06	Down	desaturase delta 9 desaturase	FADS9

[a] Log_2 fold change between P- and P+ conditions based on RNA-seq data. Each value is the mean from three biological replicates.

* Significance of change is set at the absolute value of log_2 FC > 1 and adjusted p-value < 0.05 in this study.

[-] No change in transcriptional level.

Supplementary Table 8. Responses of SPX, PHR and their ratio to P deficiency.

Gene Name	Gene ID	Expression under P+ (FPKM)	Expression under P- (FPKM)	Statistics
SPX	Phatr3_J47434	121.57	932.99	p<0.05
PHR	Phatr3_J47256	14.05	255.66	p<0.05
Ratio of PHR to SPX		1:9	1:4	p<0.05

Supplementary Table 9. The information of ribosome biogenesis related genes in *m*SPX/WT comparison under P+ condition of *P. tricornutum*.

GeneID	Log_2 FC[a]	adjusted p-value	Up/Down	Description
Phatr3_J28359	1.03	1.17E-08	Up*	60S ribosomal protein L10a
Phatr3_J15632	1.04	2.48E-08	Up*	60S acidic ribosomal protein-domain-containing protein
Phatr3_J48950	1.49	7.14E-35	Up*	60S ribosome subunit biogenesis NIP7-like protein
Phatr3_J27851	1.28	2.97E-12	Up*	small subunit ribosomal protein S10e
Phatr3_J28979	1.19	2.13E-12	Up*	large subunit ribosomal protein L12e
Phatr3_J43162	1.06	5.06E-12	Up*	large subunit ribosomal protein L27Ae
Phatr3_J19025	1.24	1.42E-13	Up*	RL9, ribosomal protein 9

[a] Log_2 fold change between P- and P+ conditions based on RNA-seq data. Each value is the mean from three biological replicates.

* Significance of change is set at the absolute value of log_2 FC > 1 and adjusted p-value < 0.05 in this study.

Supplementary Table 10. Primers used for identifying transformed strains and for RT-qPCR validation of RNA-seq data.

Primer Name	Primer sequence (5'-3')<br>Forward/Reverse	Application	Amplicon length (bp)
Cas9_identify	CGAAGTCAGAGCAGGAAATTGG<br>TCGTTTCCCTTCTGGAGTTCG	Gene identifying	607
*m*SPX_identify	CTCACCGTATTGCTGATTG<br>AGGACCATTTCTCATTCACC	Mutation region	473
PhoA (Phatr3_J49678)	GAACCATGTTTGACGTTTCGG<br>AAGGTAGTGGTCGTGGTCGG	RT-qPCR	113
PhoD (Phatr3_J45757)	GCCATTTATGCCGACACCT<br>TCCGATAGGCAGGCACATT	RT-qPCR	130
PT (Phatr3_J23830)	CAGGATGGTGTTGTTGAG<br>CGAGTCAGTTTGTAGCCG	RT-qPCR	131
PT (Phatr3_J47239)	CTTCTTCTACGACGACCTC<br>CCATCTGACCCATAGCAAC	RT-qPCR	135
PT (Phatr3_J47666)	CACTTGTCTTGCCGGTCTTG<br>ACCAACCAAAGGAGTCAACG	RT-qPCR	178
GDPD (Phatr3_J32057)	CTTTGCCAACTGTATGGGTG<br>GAGATTGTGATGGTGGCTAC	RT-qPCR	120
GDPD (Phatr3_J49693)	CTTCAAGTACGCTCAGACGC<br>TTCCACTCCAAACTGGTYGG	RT-qPCR	115
ENTPPL (Phatr3_J52110)	CGTTTGTGTGCTCCGTTCTA<br>CCTTAAGGCATTGGAGTCACTC	RT-qPCR	188
SPX (F1/R1) (Phatr3_J47434)	GCTCAATCGTAGTCCTGGTG<br>ACAAGGACCATTTCTCATTC	RT-qPCR	182
SPX (F2/R2) (Phatr3_J47434)	TTGGAGATGATTAGTCGG<br>TTCTCCTTCCGCAGTATC	RT-qPCR	153
SPX (Phatr3_J50019)	ACGGGTGAGTCGGATTCG<br>CATTGTGAGTCTTTCGTTCG	RT-qPCR	169
SPX (Phatr3_J19586)	GTTCTTTACAGTTGGTGGAC<br>CATTCAGTAGTCGTCCTAAC	RT-qPCR	145
RPS (Phatr3_J10847)	CGAAGTCAACCAGGAAACCAA<br>GTGCAAGAGACCGGACATACC	RT-qPCR	166
