# Supplementary material for: SPX-related genes regulate phosphorus homeostasis in the marine phytoplankton, Phaeodactylum tricornutum
Source: Commun Biol. 2021 Jun 25;4:797. doi: 10.1038/s42003-021-02284-x (PMC8233357; doi:10.1038/s42003-021-02284-x)
Supplement: Supplementary file 3 — Description of Additional Supplementary Files [file 42003_2021_2284_MOESM3_ESM.pdf]

Description of Additional Supplementary Files

File name: Supplementary Data 1-4

Description:

Data S1 | The information of differently expressed genes (DEGs) in *P. tricornutum* in the *m*SPX_P+/WT_P+ (grey shaded) and *m*SPX_P-/WT_P- (blue shaded) comparisons.

Data S2 | The SPX domain-containing genes identified in microeukaryotes from MMETSP dataset.

Data S3 | The SPX related genes identified from MGTs dataset.

Data S4 | Source data for the graphs presented in the main figures (Fig. 1g, 2, 3) and Fig. S2.
