# Supplementary material for: SPX-related genes regulate phosphorus homeostasis in the marine phytoplankton, Phaeodactylum tricornutum
Source: Commun Biol. 2021 Jun 25;4:797. doi: 10.1038/s42003-021-02284-x (PMC8233357; doi:10.1038/s42003-021-02284-x)
Supplement: Supplementary file 5 — Reporting Summary [file 42003_2021_2284_MOESM5_ESM.pdf]

nature research

Corresponding author(s): Senjie Lin

Last updated by author(s): May 27, 2021

Reporting Summary

Nature Research wishes to improve the reproducibility of the work that we publish. This form provides structure for consistency and transparency in reporting. For further information on Nature Research policies, see our Editorial Policies and the Editorial Policy Checklist.

Statistics

For all statistical analyses, confirm that the following items are present in the figure legend, table legend, main text, or Methods section.

n/a	Confirmed	
☐	☒	The exact sample size (*n*) for each experimental group/condition, given as a discrete number and unit of measurement
☒	☐	A statement on whether measurements were taken from distinct samples or whether the same sample was measured repeatedly
☒	☐	The statistical test(s) used AND whether they are one- or two-sided<br>*Only common tests should be described solely by name; describe more complex techniques in the Methods section.*
☒	☐	A description of all covariates tested
☒	☐	A description of any assumptions or corrections, such as tests of normality and adjustment for multiple comparisons
☒	☐	A full description of the statistical parameters including central tendency (e.g. means) or other basic estimates (e.g. regression coefficient) AND variation (e.g. standard deviation) or associated estimates of uncertainty (e.g. confidence intervals)
☐	☒	For null hypothesis testing, the test statistic (e.g. *F*, *t*, *r*) with confidence intervals, effect sizes, degrees of freedom and *P* value noted<br>*Give P values as exact values whenever suitable.*
☒	☐	For Bayesian analysis, information on the choice of priors and Markov chain Monte Carlo settings
☒	☐	For hierarchical and complex designs, identification of the appropriate level for tests and full reporting of outcomes
☒	☐	Estimates of effect sizes (e.g. Cohen's *d*, Pearson's *r*), indicating how they were calculated

Our web collection on statistics for biologists contains articles on many of the points above.

Software and code

Policy information about availability of computer code

Data collection	N/A
Data analysis	Prism v8 (GraphPad), MEGA X, R version 4.0.5, Pfam, PhytoCRISP-Ex (a CRISPR target finding tool), HISAT software (http://ccb.jhu.edu/software.shtml), StringTie software (http://ccb.jhu.edu/software.shtml), DESeq2 software package (http://www. bioconductor.org/packages/release/bioc/html/DESeq2.html), HMMER hmmsearch program, SPSS software (version 16.0, IBM, US).

For manuscripts utilizing custom algorithms or software that are central to the research but not yet described in published literature, software must be made available to editors and reviewers. We strongly encourage code deposition in a community repository (e.g. GitHub). See the Nature Research guidelines for submitting code & software for further information.

Data

Policy information about availability of data

All manuscripts must include a data availability statement. This statement should provide the following information, where applicable:

- Accession codes, unique identifiers, or web links for publicly available datasets
- A list of figures that have associated raw data
- A description of any restrictions on data availability

Raw sequencing data is available at NCBI in the SRA (Short Read Archive) database under accession number SRP214503. Source data corresponding to the figures are included in Supplementary Data 4, and uncropped gel image corresponding to Fig. 1d is included in Supplementary Fig. 3.

Field-specific reporting

Please select the one below that is the best fit for your research. If you are not sure, read the appropriate sections before making your selection.

☐ Life sciences ☐ Behavioural & social sciences ☒ Ecological, evolutionary & environmental sciences

For a reference copy of the document with all sections, see nature.com/documents/nr-reporting-summary-flat.pdf

Ecological, evolutionary & environmental sciences study design

All studies must disclose on these points even when the disclosure is negative.

Study description	We reports a novel approach to address, and exciting mechanistic insights into, the regulatory cascade of phosphorus homeostasis in marine phytoplankton. Using the cutting-edge CRISPR/Cas9 genome editing tool combined with physiological measurements, we detect and provide experimental proof of a molecular mechanism (SPX-PHR) as the missing essential regulator. We further provide evidence that this mechanism is universal across the spectrum of phytoplankton and the geographic range of the world's ocean.
Research sample	Phaeodactylum tricornutum strain CCAP 1055/1 was obtained from the Culture Collection of Algae and Protozoa. The mutant lines (mSPX) were created using CRISPR/Cas9 technology. For the comparison of growth between wild type and mutant, a matrix of 4 groups were experimented separately with different phosphorous conditions: P+ (36 μM DIP) and P– (DIP < 0.5 μM), each in triplicate.
Sampling strategy	In addition to experimental samples, which were triplicated as commonly practiced in the field, this study also uses existing databases of the Marine Microbial Eukaryotic Transcriptome Sequencing Program (MMETSP), a global ocean metatranscriptomic dataset (TARA OCEANS), and the metagenomics-based transcriptomes (MGTs) to cover as wide phylogenetic range of phytoplankton and global ocean range as possible
Data collection	Transcriptome sequencing was conducted using Illumina (RNA-seq). The alignment of our transcriptome reads to the genome was performed using the HISAT2 software. StringTie and DESeq2 software were used to estimate transcript abundances and identify differentially expressed genes among different groups (FDR < 0.05, fold change > 2), respectively.
Timing and spatial scale	Global ocean sampling data was done by TARA Oceans expedition, which we used for analysis.
Data exclusions	*If no data were excluded from the analyses, state so OR if data were excluded, describe the exclusions and the rationale behind them, indicating whether exclusion criteria were pre-established.*
Reproducibility	*Describe the measures taken to verify the reproducibility of experimental findings. For each experiment, note whether any attempts to repeat the experiment failed OR state that all attempts to repeat the experiment were successful.*
Randomization	*Describe how samples/organisms/participants were allocated into groups. If allocation was not random, describe how covariates were controlled. If this is not relevant to your study, explain why.*
Blinding	*Describe the extent of blinding used during data acquisition and analysis. If blinding was not possible, describe why OR explain why blinding was not relevant to your study.*

Did the study involve field work? ☐ Yes ☒ No

Reporting for specific materials, systems and methods

We require information from authors about some types of materials, experimental systems and methods used in many studies. Here, indicate whether each material, system or method listed is relevant to your study. If you are not sure if a list item applies to your research, read the appropriate section before selecting a response.

Materials & experimental systems

n/a	Involved in the study	
☒	☐	Antibodies
☒	☐	Eukaryotic cell lines
☒	☐	Palaeontology and archaeology
☒	☐	Animals and other organisms
☒	☐	Human research participants
☒	☐	Clinical data
☒	☐	Dual use research of concern

Methods

n/a	Involved in the study	
☒	☐	ChIP-seq
☒	☐	Flow cytometry
☒	☐	MRI-based neuroimaging

April 2020
